# Assessment of Diabetes Self-Care Practices Among Patients at a Military Hospital in Saudi Arabia: A Cross-Sectional Study

**DOI:** 10.7759/cureus.97125

**Published:** 2025-11-17

**Authors:** Ghadeer Hassounah, Reem Qomsani, Asirvatham Alwin Robert

**Affiliations:** 1 Department of Health Education, Prince Sultan Military Medical City, Riyadh, SAU; 2 Department of Endocrinology and Diabetes, Prince Sultan Military Medical City, Riyadh, SAU

**Keywords:** diabetes self-care, diabetes self-management questionnaire, glycemic control, patient education, saudi arabia, type 1 and type 2 diabetes

## Abstract

Aim: This study aimed to evaluate diabetes self-care behaviors among patients with type 1 and type 2 diabetes attending Prince Sultan Military Medical City, Riyadh, Saudi Arabia, using the Diabetes Self-Management Questionnaire (DSMQ).

Methods: A cross-sectional observational study was conducted between June and September 2025. Adults (≥18 years) with a confirmed diabetes diagnosis for at least six months were recruited, excluding those with severe psychiatric conditions, cognitive impairment, or incomplete responses. Participants completed the DSMQ and a demographic/clinical questionnaire. The DSMQ evaluates four domains: glucose management, dietary control, physical activity, and healthcare use, with higher scores indicating better self-management.

Results: Among 130 participants, 53.1% were female, and 58.5% were aged <40 years. Type 1 and type 2 diabetes were nearly evenly represented. Most participants had a body mass index* (*BMI) ≥25 kg/m^2^ and suboptimal glycemic control (HbA1c ≥7%). Overall, DSMQ scores were moderate, indicating room for improvement. Physical activity scores were slightly higher in type 2 diabetes (p = 0.042), while other subscales and total scores showed no significant differences across diabetes type, age, gender, BMI, duration, or HbA1c levels. Higher BMI was associated with increased healthcare engagement (p = 0.023).

Conclusion: Self-management behaviors among this cohort were generally consistent across demographic and clinical subgroups, with modest differences in physical activity and healthcare use. These findings underscore the need for targeted educational interventions to enhance diabetes self-care, optimize glycemic control, and reduce complications in the Saudi population.

## Introduction

Diabetes mellitus (DM) is a major global health concern, widely regarded as one of the most serious long-term illnesses because of its rising prevalence and the severe complications it brings [1,2]. Over the past three decades, the worldwide incidence of diabetes has increased sharply, affecting people across all economic and social groups. According to the International Diabetes Federation (IDF), an estimated 537 million adults between the ages of 20 and 79 currently live with the condition, and this number is projected to climb to 784 million by 2045 [3]. Such growth highlights the heavy toll diabetes exerts on individuals, families, and healthcare systems, making it crucial to develop effective measures to control the disease and lessen its social and economic consequences.

In Saudi Arabia, diabetes poses a particularly serious challenge, with nearly one in four adults currently affected. Projections indicate that this rate could more than double by 2030 [4-6]. Cases of type 1 diabetes (T1D) are also increasing steadily, with an annual rise of about 3-4%, and diagnoses are occurring in children at progressively younger ages. Globally, Saudi Arabia ranks seventh in the prevalence of T1D and fifth in incidence, although the underlying causes behind these figures are still not fully understood [7,8]. The marked growth of both T1D and type 2 diabetes (T2D) underscores the pressing need for robust public health initiatives and patient-focused strategies to improve diabetes prevention and management within the nation.

Successful diabetes management relies heavily on consistent self-care, which involves taking prescribed medications correctly, monitoring blood sugar levels, maintaining a healthy diet, engaging in regular physical activity, and staying in contact with healthcare professionals [9]. Assessing these practices is vital to pinpoint shortcomings in patient knowledge, daily routines, and resource accessibility. Gaining insight into how individuals manage their condition on a daily basis allows healthcare providers to create personalized interventions, strengthen patient education, and improve both blood glucose control and quality of life [10,11]. Research consistently shows that effective self-care is strongly linked to improved disease outcomes and reduced complications, underscoring its central role in diabetes care [12].

The Diabetes Self-Management Questionnaire (DSMQ) is a widely recognized tool designed to evaluate participation in key aspects of diabetes self-care, such as blood glucose monitoring, dietary practices, physical activity, and engagement with healthcare services [13]. Examining patient responses through this instrument helps researchers detect behavioral trends that affect diabetes management and uncover factors influencing treatment adherence [13]. The present study investigates self-care behaviors among individuals receiving care at the Diabetes Treatment Center of Prince Sultan Military Medical City in Riyadh, Saudi Arabia. Findings from this work are expected to guide the creation of tailored educational strategies and interventions to strengthen self-management and enhance health outcomes within this patient group.

## Materials and methods

This study was conducted as an analytical cross-sectional study to assess self-care behaviors among individuals with diabetes using the DSMQ. The study population included patients with T1D and T2D who were receiving routine care at Prince Sultan Military Medical City, Riyadh, Saudi Arabia.

Inclusion and exclusion criteria

Adults aged 18 years and above with a confirmed diagnosis of either T1D (n=63) or T2D (n=66) for a minimum of six months were considered eligible for inclusion. Participants needed to be literate in Arabic and willing to provide informed consent. Individuals with gestational diabetes, significant cognitive or psychiatric disorders that could hinder reliable responses, as well as those who were critically ill or hospitalized during data collection, were excluded. Upon giving consent, participants were asked to complete the DSMQ in addition to providing demographic and clinical information.

Ethical approval

The research protocol received approval from the Institutional Review Board (IRB) at Prince Sultan Military Medical City, Riyadh, Saudi Arabia (approval no. E-2586). Prior to participation, all individuals signed written informed consent forms. The study was carried out in line with the ethical standards of the Declaration of Helsinki as well as local and institutional regulations for human research. To safeguard privacy, participant information was kept confidential, and all collected data were anonymized.

Data collection

Demographic and clinical characteristics of the study population were collected, including gender, age, diabetes type, duration of diabetes, body mass index (BMI), HbA1c levels, and insulin pump use. Comparisons of DSMQ subscale and total scores were made across different participant subgroups, including type of diabetes, age categories, duration of diabetes, gender, BMI categories, and HbA1c levels.

The Diabetes Self-Management Questionnaire (DSMQ)

The DSMQ was employed to evaluate self-care practices among individuals with diabetes [13]. It contains 16 items, each rated on a four-point Likert scale ranging from 0 (“does not apply”) to 3 (“applies very much”), covering behaviors over the previous eight weeks. These items are divided into four domains: Glucose Management (items 1, 4, 6, 10, and 12; items 10 and 12 reverse-scored), Dietary Control (items 2, 5, 9, and 13; items 5 and 13 reverse-scored), Physical Activity (items 8, 11, and 15; items 11 and 15 reverse-scored), and Health-Care Use (items 3, 7, and 14; items 7 and 14 reverse-scored). The final item (16) is reverse-coded and contributes only to the overall score. For analysis, negatively phrased items were recoded using the formula (3 - original response). Subscale scores were obtained by dividing the total of the recoded items by the maximum possible value (number of items × 3), then converting the result to a 0-10 scale. Higher values indicate stronger adherence to self-care, with the overall DSMQ score (Sum Scale) calculated from all 16 items, yielding a maximum possible score of 10 [13-15].

Statistical analysis

All statistical analyses were performed using IBM SPSS Statistics for Windows, Version 25 (Released 2017; IBM Corp., Armonk, New York, United States). Descriptive statistics were used to summarize the demographic and clinical characteristics of the study participants, with categorical variables presented as frequencies and percentages, and continuous variables expressed as means and standard deviations (SD). Independent sample t-tests were applied to compare DSMQ subscale and total scores between two-group variables, such as gender, type of diabetes, and age category (<40 years vs. ≥40 years). The association between diabetes duration (<7 years vs. ≥7 years) and DSMQ scores was also examined using t-tests. All negatively worded items were reverse-coded prior to analysis to ensure consistency of scoring. Statistical significance was set at a p-value <0.05.

## Results

Table 1 summarizes data from 130 participants: 53.1% were female, 58.5% were under 40 years, and 51.2% had T2D. Diabetes duration was under 10 years in 56.9%. Overweight/obesity (BMI ≥25) affected 64.6%, poor glycemic control (HbA1c ≥7%) was seen in 88.5%, and only 3.1% used an insulin pump.

**Table 1:** Demographic and Clinical Characteristics of the Study Population

Variables	Frequency (%)
Gender	
Female	69 (53.1)
Male	61 (46.9)
Age	
<40 years	76 (58.5)
≥40 years	54 (41.5)
Diabetes types	
Type 1 diabetes	63 (48.8)
Type 2 diabetes	66 (51.2)
Duration of diabetes	
<10 years	74 (56.9)
≥10 years	56 (43.1)
BMI (kg/m^2^)	
<25 years	46 (35.4)
≥25 years	84 (64.6)
HbA1c	
<7 years	15 (11.5)
≥7 years	115 (88.5)
Using an insulin pump	
Yes	4 (3.1)
No	126 (96.9)

Table 2 shows similar DSMQ scores for males and females in glucose management, diet, and health care. Males had slightly higher physical activity (6.2 vs. 3.8) and total scores (6.3 vs. 5.5), indicating marginally better self-management.

**Table 2:** Comparison of DSMQ Subscale Scores by Gender A p-value < 0.05 was considered statistically significant. DSMQ: Diabetes Self-Management Questionnaire

Subscales of the DSMQ	Gender	Mean ± SD	t value	p-value
Glucose management	Female	6.6 ± 2.5	-0.211	0.331
	Male	6.7 ± 2.7	-0.210	
Dietary control	Female	3.9 ± 1.1	-0.475	0.898
	Male	4.0 ± 1.1	-0.474	
Physical activity	Female	3.8 ± 2.7	-4.59	0.224
	Male	6.2 ± 3.0	-4.56	
Heath care	Female	6.6 ± 2.3	-0.407	0.451
	Male	6.8 ± 2.1	-0.409	
Sum scale	Female	5.5 ± 1.6	-2.64	0.069
	Male	6.3 ± 1.9	-2.62	

Table 3 shows no significant age differences in DSMQ scores. Participants <40 and ≥40 years had similar scores across all subscales and total DSMQ (p > 0.05).

**Table 3:** Comparison of DSMQ Subscale Scores by Age Groups A p-value < 0.05 was considered statistically significant. DSMQ: Diabetes Self-Management Questionnaire

Subscales of the DSMQ	Age	Mean ± SD	t value	p-value
Glucose management	<40 yrs.	6.7 ± 2.6	0.078	0.92
	≥40 yrs.	6.69 ± 2.5	0.078	
Dietary control	<40 yrs.	3.86 ± 1.0	-1.74	0.569
	≥40 yrs.	4.21 ± 1.1	-1.72	
Physical activity	<40 yrs.	5.19 ± 3.2	1.00	0.457
	≥40 yrs.	4.63 ± 3.0	1.01	
Heath care	<40 yrs.	6.63 ± 2.1	-0.628	0.182
	≥40 yrs.	6.89 ± 2.4	-0.618	
Sum scale	<40 yrs.	5.90 ± 1.7	-0.166	0.497
	≥40 yrs.	5.95 ± 1.9	-0.164	

Table 4 compares DSMQ scores by diabetes type. Type 1 showed slightly higher glucose management (7.1 vs. 6.2) and health care (7.0 vs. 6.4), while dietary control was identical (4.0). Physical activity was marginally higher in type 2 (4.9 vs. 4.8; p = 0.042). Total scores were similar (6.0 vs. 5.7). Overall, self-management was comparable across groups.

**Table 4:** Comparison of DSMQ Subscale Scores Between Type 1 and Type 2 Diabetes A p-value < 0.05 was considered statistically significant. DSMQ: Diabetes Self-Management Questionnaire

Subscales of the DSMQ	Type of DM	Mean ± SD	t value	p-value
Glucose management	Type 1 diabetes	7.1 ± 2.7	1.75	0.13
	Type 2 diabetes	6.2 ± 2.5	1.75	
Dietary control	Type 1 diabetes	4.0 ± 1.1	0.092	0.732
	Type 2 diabetes	4.0 ± 1.1	0.092	
Physical activity	Type 1 diabetes	4.8 ± 3.4	-0.178	0.042
	Type 2 diabetes	4.9 ± 2.8	-0.178	
Heath care	Type 1 diabetes	7.0 ± 2.2	1.34	0.569
	Type 2 diabetes	6.4 ± 2.3	1.34	
Sum scale	Type 1 diabetes	6.0 ± 1.8	0.726	0.876
	Type 2 diabetes	5.7 ± 1.8	0.726	

Table 5 compares DSMQ scores by diabetes duration. Participants with <10 and ≥10 years showed similar scores across all subscales and total DSMQ (6.1 vs. 5.6; p = 0.810), indicating no difference in self-management by duration.

**Table 5:** Comparison of DSMQ Subscale Scores by Duration of Diabetes A p-value < 0.05 was considered statistically significant. DSMQ: Diabetes Self-Management Questionnaire

Subscales of the DSMQ	Duration of diabetes	Mean ± SD	t value	P Value
Glucose management	<10 yrs.	6.8 ± 2.5	0.843	0.652
	≥10 yrs.	6.4 ± 2.7	0.838	
Dietary control	<10 yrs.	3.9 ± 1.1	-0.871	0.875
	≥10 yrs.	4.1 ± 1.1	-0.869	
Physical activity	<10 yrs.	5.5 ± 3.2	2.34	0.226
	≥10 yrs.	4.2 ± 2.9	2.37	
Heath care	<10 yrs.	6.9 ± 2.2	1.19	0.847
	≥10 yrs.	6.4 ± 2.3	1.19	
Sum scale	<10 yrs.	6.1 ± 1.8	1.55	0.810
	≥10 yrs.	5. 6 ± 1.8	1.55	

Table 6 shows DSMQ scores by BMI. Participants with BMI <25 and ≥25 kg/m^2^ had similar glucose management, dietary control, physical activity, and total DSMQ scores. Health care scores were slightly higher in those with BMI ≥25 (p = 0.023).

**Table 6:** Comparison of DSMQ Subscale Scores by BMI Categories A p-value < 0.05 was considered statistically significant. DSMQ: Diabetes Self-Management Questionnaire

Subscales of the DSMQ	BMI (kg/m^2^)	Mean ± SD	t value	p-value
Glucose management	<25	6.6 ± 2.5	-0.239	0.434
	≥25	6.7 ± 2.6	-0.243	
Dietary control	<25	4.0 ± 1.2	0.378	0.809
	≥25	3.9 ± 1.1	0.367	
Physical activity	<25	5.4 ± 3.0	1.21	0.997
	≥25	4.7 ± 3.1	1.22	
Heath care	<25	6.5 ± 2.0	-0.640	0.023
	≥25	6.8 ± 3.5	-0.669	
Sum scale	<25	5.8 ± 1.8	-0.248	0.853
	≥25	5.9 ± 1.8	-0.247	

Table 7 shows DSMQ scores by HbA1c levels. Participants with HbA1c <7% and ≥7% had similar subscale and total DSMQ scores, indicating no major differences in self-management behaviors by glycemic control.

**Table 7:** Comparison of DSMQ Subscale Scores by HbA1c Levels A p-value < 0.05 was considered statistically significant. DSMQ: Diabetes Self-Management Questionnaire

Subscales of the DSMQ	HbA1c level	Mean ± SD	t value	p-value
Glucose management	<7 yrs.	6.8 ± 2.6	0.274	0.621
	≥7 yrs.	6.6 ± 2.64	0.272	
Dietary control	<7 yrs.	3.8 ± 1.36	-0.421	0.336
	≥7 yrs.	4.0 ± 1.12	-0.362	
Physical activity	<7 yrs.	5.5 ± 2.84	0.782	0.481
	≥7 yrs.	4.8 ± 3.18	0.853	
Heath care	<7 yrs.	7.1 ± 2.29	0.662	0.589
	≥7 yrs.	6.6 ± 2.28	0.660	
Sum scale	<7 yrs.	6.2 ± 1.97	0.772	0.619
	≥7 yrs.	5.8 ± 1.83	0.728	

## Discussion

This research examined self-care behaviors related to diabetes among Saudi patients with T2D and T2D, utilizing the DSMQ. Results showed that self-management practices were generally similar across different demographic and clinical groups, with only slight differences observed [14]. Patients with T2D tended to participate more in physical activity, whereas those with higher BMI demonstrated slightly greater engagement with healthcare services. These findings align with previous studies supporting the DSMQ as a reliable instrument for assessing diabetes self-care and its connection to glycemic control [13-15]. Overall, the cohort’s average DSMQ scores were moderate, highlighting the importance of targeted interventions to strengthen diabetes management.

When analyzed according to diabetes type, patients with T2D exhibited higher physical activity scores compared with those having T1D. This contrasts with previous studies suggesting that T1D often engage in more physical activity due to lifestyle-focused management strategies [16,17]. The discrepancy may be influenced by cultural and environmental factors specific to Saudi Arabia, where opportunities for structured exercise might be limited, particularly for younger individuals with T1D. Additionally, clinical practices in the region often place greater emphasis on lifestyle counseling for T2D patients, which may contribute to the observed differences in activity levels.

When analyzed by gender and age, no notable variations were observed in DSMQ scores. Men, however, reported marginally higher engagement in physical activity, aligning with earlier research suggesting that men tend to be more active in exercise-related self-care, whereas women often show stronger adherence to dietary and medication routines [18,19]. In this study, dietary practices appeared consistent between men and women, possibly reflecting the impact of structured education programs in specialized healthcare settings, which may help minimize such differences. Likewise, age-related variations were minimal, contrasting with previous reports that younger patients often struggle more with diet and glucose monitoring [20]. These outcomes may reflect the positive association of ongoing diabetes education initiatives within this population.

In terms of clinical features, BMI and HbA1c values had minimal effect on most DSMQ domains. The only notable finding was that individuals with higher BMI reported greater use of healthcare services, likely due to the need for managing comorbidities and receiving more frequent medical supervision [21,22]. No significant relationship was observed between HbA1c and DSMQ scores, which may be explained by the overall high prevalence of poor glycemic control in the study population, limiting score variability [23]. However, earlier studies have highlighted associations between DSMQ outcomes and glycemic control [13]. Further research with larger and more diverse Middle Eastern cohorts is needed. Longitudinal studies could also provide deeper insights into how DSMQ-assessed self-care practices affect long-term health outcomes and guide culturally tailored interventions.

Limitations

This study is subject to certain limitations. To begin with, the cross-sectional approach limits the ability to determine cause-and-effect relationships between self-care behaviors and glycemic control, as information was gathered at only one point in time. Long-term cohort studies would provide clearer insights into how self-management influences disease progression and complications. Additionally, the investigation was restricted to a single tertiary military hospital in Riyadh, which may reduce the applicability of the results to the wider Saudi population, particularly those in rural areas or under different healthcare systems. Another limitation lies in the use of self-reported data via the DSMQ, which is prone to recall errors and social desirability bias, potentially leading participants to misrepresent their actual practices. These constraints emphasize the importance of conducting future studies with multi-center participation, the use of objective assessment tools, and longitudinal follow-up to achieve a more thorough understanding of diabetes self-management across Saudi Arabia.

## Conclusions

In summary, the findings of this study indicate that diabetes self-care behaviors in Saudi Arabia, as measured by the DSMQ, remain at a moderate level, leaving room for further improvement. Patients with T2D reported greater involvement in physical activity, likely due to the emphasis on lifestyle guidance during routine care. Differences by gender and age were minimal, suggesting that structured education programs may help reduce disparities across these groups. Individuals with higher BMI engaged more frequently with healthcare services, underscoring the importance of providing targeted support for overweight and obese patients. No meaningful link was found between HbA1c and DSMQ scores, which may be attributed to the widespread presence of inadequate glycemic control in the study cohort. Overall, the results affirm the value of the DSMQ as an effective instrument for assessing diabetes self-management in this setting, while emphasizing the need for longitudinal research to inform culturally appropriate interventions aimed at strengthening self-care and improving clinical outcomes.
